# CT measurement of prostate volume using OsiriX^®^ viewer is reliable, repeatable, and not dependent on observer, CT protocol, or contrast enhancement in dogs

**DOI:** 10.1111/vru.13125

**Published:** 2022-07-05

**Authors:** Hanna M. Salonen, Tuuli M. Åhlberg, Outi M. Laitinen‐Vapaavuori, Sari H. Mölsä

**Affiliations:** ^1^ Department of Equine and Small Animal Medicine Faculty of Veterinary Medicine University of Helsinki Helsinki Finland

**Keywords:** canine, intraobserver, interobserver

## Abstract

Computed tomography (CT) is an established method for evaluating dogs with suspected prostatic disease; however, publications assessing the effects of varying factors on prostate volume measurements are lacking. The objectives of this two‐part, observer agreement, methods comparison study were to assess observer agreement and the effects of varying CT technical parameters for volume measurements of canine prostate glands on CT images using OsiriX^®^ DICOM viewer software. In the first retrospective study, two observers measured prostate volumes of 13 client‐owned dogs thrice on noncontrast and contrast CT images. In the second prospective study, two observers measured the prostate volume of 10 cadavers using five different CT protocols and eight cadavers using three slice thicknesses. Observer agreement analyses were performed, and prostatic CT volume measurements were compared with water displacement volume measurements. Intra‐ and interobserver variability and the effect of contrast enhancement were found to be minimal when a one‐way analysis of variance model and intraclass correlation coefficients were used. No significant differences emerged between different protocols and slice thicknesses using a linear mixed effects model. When the prostate CT volume was compared using a Bland–Altman plot with the reference volume acquired by the water displacement method, agreement without consistent bias between the methods was shown, and over 90% of measurements were located within the 95% limits of agreement. The findings supported using OsiriX^®^ software for CT prostatic volume measurements in dogs.

**Abbreviations**
ANOVA: analysis of variance
CI: confidence interval
DICOM: digital imaging and communication in medicine
ICC: intraclass correlation coefficient
LoA: limits of agreement
ROI: region of interest
VTHUH: Veterinary Teaching Hospital of the University of Helsinki

## 1 INTRODUCTION

Prostate diseases are common among intact male dogs and often lead to prostate gland enlargement.^1^, ^2^, ^3^, ^4^, ^5^ Traditionally, the size of the prostate gland has been evaluated using subjective assessments such as rectal palpation or radiographic or ultrasonographic imaging.^4^, ^5^, ^6^, ^7^, ^8^ Recently, computed tomography (CT) has become widely available in small animal practice. Several studies on dogs have shown CT imaging to be a reliable tool for investigating prostate gland structure and size.^9^, ^10^, ^11^, ^12^, ^13^ Prostate gland boundaries, lesions, location, and surrounding tissues are more visible in CT than with ultrasonographic imaging, allowing more exact measurements to be taken.^9^, ^10^, ^11^, ^12^, ^13^ Earlier, one‐dimensional parameters, such as length, width, and height, have been measured; later, prostate volume has been calculated by advanced software.^9^, ^10^, ^11^, ^12^, ^13^ Accurate volume measurements allow development of reference value ranges and serve as an objective tool for characterizing severity of prostatomegaly.

Computed tomography volumes of objects and organs in both dogs and humans have been compared with reference volumes, with actual volumes determined by the water displacement method^12^, ^14^, ^15^ or known water content,^12^, ^15^, ^16^ or with resection weights.^17^ Computed tomography volume measurements have recently reached an accuracy of ±0.8%,^12^ compared with ±5%^14^ to ±10%^15^ in the early 1980s. The number of different DICOM viewer software programs available for assessing CT images is extensive, and currently, many software programs have specific tools for CT volume measurements. Many of these have been used in volume measurement studies of multiple organs with or without reference methods. For example, Amira^®^ software has been used to measure the CT volume of the canine^12^ (version 6.2, Hillsboro, Oregon, USA) and human prostate^20^ (version 3.1, Berlin, Germany), and OsiriX^®^ (Geneva, Switzerland) software has been used for measurements of the human liver^17^, ^18^ and orbita.^19^ Based on our review of previous literature, no published studies described measuring canine prostate volume using OsiriX^®^ and compared it with a reference standard method. No studies were found in which intra‐ or interobserver variability of canine prostate CT volume measurement had been analyzed. Although technical parameters of CT scans might have an impact on volume measurements, no published studies have assessed the impact of tube current (mA), tube potential (kV), imaging algorithm, window level, or contrast enhancement on volume measurements. Two previous studies evaluated the effect of slice thickness and found that smaller slice thicknesses gave more accurate results.^20^, ^21^ While parameters can be kept constant in prospective studies, this is often not possible in multicenter and retrospective studies.

The objectives of our two‐part study were to evaluate the intra‐ and interobserver variability using two observers with different experience levels as well as the effect of different CT protocols and use of contrast agent on volume measurements of prostates of intact male dogs in CT using the OsiriX^®^ volume measurement tool. Additionally, we aimed to compare the CT volumes of the prostates with actual reference volumes. Our hypothesis was that CT volume measurements using the OsiriX^®^ volume measurement tool would be reliable, repeatable, and not influenced by observer, CT protocol, or contrast medium.

## 2 MATERIALS AND METHODS

This was a single‐center, two‐part, observer agreement and method comparison study performed at the Veterinary Teaching Hospital of the University of Helsinki (VTHUH).

### 2.1 Sample population

**FIGURE 1:**
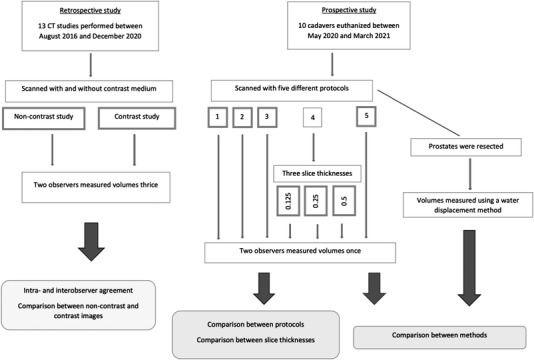
Flow chart diagram illustrating how measurements for this study were implemented. Boxes with bold margins indicate the image series that were used for measurements

In the first retrospective study, the patient data were collected from the VTHUH database. Client‐owned intact male dogs aged 5 years or older that had undergone a CT scan of the caudal abdomen from August 2016 to December 2020 were included in the study (Figure 1). Only scans including the entire prostate in both noncontrast and contrast images were considered. Dogs with known prostatic disease, hormonal implants, or treatment were excluded.

In the second prospective study, CT scans of the caudal abdomen, including the entire prostate, were performed on client‐owned intact male dogs that had been euthanized at VTHUH during the period from May 2020 to March 2021 (Figure 1). As in the retrospective study, dogs with known prostatic disease, hormonal implants, or treatment were excluded. Before anonymization, decisions for including and excluding dogs for both studies were made by one of the observers (H.M.S.) with 12 years of experience in veterinary diagnostic imaging.

### 2.2 Ethical considerations

In the retrospective study, based on Finnish national legislation, the need for ethical approval or owners’ consent was deemed unnecessary since data were anonymized before analysis (https://finlex.fi/en/laki/kaannokset/2013/20130497). In the prospective study, owners gave consent according to the VTHUH policy for the use of cadavers for research and teaching purposes.

### 2.3 CT imaging protocols

As part of the inclusion criteria for the study, all CT scans were performed with a 64‐slice helical MDCT scanner (Lightspeed VCT^®^, GE Healthcare, Madison, WI, USA). The CT data were stored in DICOM format in the VTHUH's picture archiving and communications system (PACS). In both parts of the study, the detector coverage was 40.0 mm. A field of view was selected according to dog or cadaver size, and the matrix was 512 × 512. Transverse images were acquired in dorsal recumbency. An automatic current selection was used.

In the prospective study, all cadavers underwent CT scans with five different protocols chosen to represent scans of the retrospective study and those that are routinely used in VTHUH. Slice and interval thicknesses of 2.5 mm were used in one protocol and 0.625 mm in the others. The tube potential was 120 kV. The collimation pitch was 0.516, 0.984, or 1.375:1, speed 20.62, 39.37, or 55.00 mm/rotation, and rotation time 0.4–0.8 s. The tube current varied between 80 and 697 mA depending on the cadaver's size and the noise index. The noise index varied between 10 and 20. Technical parameters of the CT scans concerning specific protocols are presented in Table 1.

**TABLE 1:** Technical parameters of five CT protocols used in the scans of ten cadavers’ caudal abdomen in the prospective study

Protocol #	Slice thickness (mm)	Voltage (kV)	Pitch	Speed (mm/rot)	Rotation time (s)	Noise index
1	0.625	120	0.984:1	39.37	0.4	18.00
2	0.625	120	0.516:1	20.62	0.6	20.00
3	0.625	120	0.984:1	39.37	0.5	11.05
4	0.625	120	0.516:1	20.62	0.8	10.00
5	2.5	120	1.375:1	55.00	0.5	20.00

### 2.4 CT volume measurements

All measurements were performed by two observers, observer #1 (H.M.S.) and #2 (T.M.Å.). Both were licensed veterinarians, the former with 12 years of experience in veterinary diagnostic imaging and the latter a PhD student in clinical veterinary medicine familiarized with the use of OsiriX^®^ and performing CT measurements. Observers were blinded to the clinical histories of the dogs and cadavers, the results of their own previous measurements, and the results of other observers’ measurements. The time between each measurement of one dog was a minimum of two days, when repeated measurements for intraobserver evaluation were performed.

**FIGURE 2:**
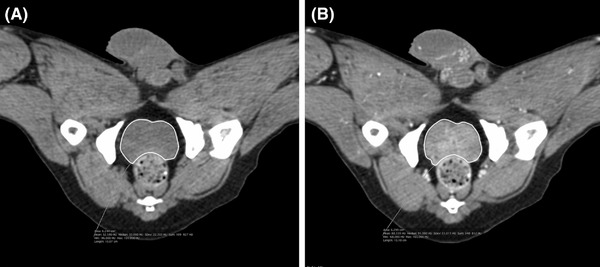
Transverse noncontrast (A) and contrast (B) computed tomography images of the pelvic area of the same dog in dorsal recumbency demonstrating prostate margins traced on images with a freehand image tracing software tool. Computed tomographic images were reconstructed with a soft tissue algorithm. A slice thickness of 0.625 mm, window level of 40 HU, and window width of 400 HU were used

Images were transferred to a workstation using commercially available DICOM viewer software (OsiriX^®^, version 11.0.4, Pixmeo, Switzerland). Image series reconstructed with a soft tissue algorithm were selected for all analyses. Volume measurements were performed using standardized soft tissue window settings (window level 40 HU; window width 400 HU). Prostate margins were drawn on every sequential transverse image with a freehand image tracing software tool (Closed Polygon; Figure 2) as regions of interest (ROIs). After selecting all of the ROIs within one series, prostatic volume was calculated using another software tool, Compute Volume. OsiriX^®^ automatically calculated the volume by multiplying surface and slice thickness and then adding up individual slice volumes.^17^ The results were displayed with four decimal places in a separate window with a 3D rendered view of the volume ROI.

In the retrospective study, two observers measured prostate volumes thrice from both noncontrast and contrast image series. In the prospective study, two observers measured prostate volumes once from five separate image series of each cadaver scanned with different CT protocols. Additionally, two extra reconstructions with slice and interval thicknesses of 1.25 and 2.5 mm were calculated from the raw data of the image series scanned with protocol #4. Prostate volumes from these extra reconstructions were measured once by both observers (Figure 1).

### 2.5 Water displacement volume measurements

In the prospective study, the prostates were carefully resected from the cadavers immediately after CT scanning, and their volumes were measured thrice by observer #1 (Figure 1) using a previously described water displacement method.^8^, ^12^, ^14^, ^15^ The water displacement method consisted of placing the prostate in a graduated cylinder containing a recorded volume of water, after which the displaced volume of water was recorded. The size of the cylinder (with 1–5 cm^3^ steps) was optimized for the size of the prostate.

### 2.6 Statistical methods

Statistical tests were selected and completed by a statistician. All statistical analyses were performed using commercially available software (SAS^®^ System for Windows, version 9.4, SAS Institute Inc., Cary, NC, USA). Descriptive statistics are presented as average ± SD (range) values of the continuous variables.

In the retrospective study, intra‐ and interobserver agreement was assessed to evaluate the repeatability of the prostate volume measurements. The noncontrast and contrast image series were evaluated separately as noncontrast and contrast groups. The random variation caused by repeating measures within an observer was assessed in two different ways. First, the repeatability between the repeats was calculated with a one‐way analysis of variance (ANOVA) model, where the effect of dog was used as a fixed effect. In these models, the within‐group variation described the variation between the repeats. The presented values were considered a percentage of perfect agreement. Second, to determine the intraobserver reliability estimate between the repeats, intraclass correlation coefficients (ICCs) with 95% confidence intervals (CIs) were calculated to assess consistency between the repeats. ICC was calculated both within observers and for combined data, including both observers (within noncontrast or contrast groups), and finally for the full data, including both observers and image series. ICC values of 0.01–0.2 were considered to have “slight agreement,” 0.21–0.40 “fair agreement,” 0.41–0.60 “moderate agreement,” 0.61–0.80 “substantial agreement,” and 0.80–1.00 “almost perfect agreement.”^22^ The random variation between observers (interobserver reliability) was evaluated with the average values of each observer using a similar methodology as described for the intraobserver repeatability.

In the prospective study, statistical analyses were performed between volume measurements from images obtained using five different protocols, between three different slice thicknesses, and between two different methods, i.e., CT volumetry and water displacement method. The different protocols to measure the prostate volume were compared with each other using a linear mixed effects model. The model included the protocol as the sole fixed effect and observer and dog as the random effects. Compound symmetry was used as the covariance structure. The agreement of the two observers, regardless of the protocol, was assessed with a scatter plot and by evaluating the regression equation based on a simple linear regression analysis between the two observers, where zero as the intercept and one as the slope would mean identical results between the observers. In addition, the five protocols were compared pairwise against the water displacement method using Bland–Altman plots. Based on the observed average difference, 95% limits of agreement (LoA) were constructed. The three slice thicknesses were compared with a similar linear mixed effect model as the measurement protocols using slice thickness as the fixed effect and observer and dog as the random effects.

## 3 RESULTS

### 3.1 Sample population and image acquisition

In the retrospective study, 13 dogs met the inclusion criteria. The average ± SD (range) age and body weight of the dogs were 8.6 ± 2.7 (5.1–13.2) years and 22.6 ± 21.9 (1.6–62.5) kg, respectively. The dogs represented 12 different breeds: Yorkshire Terrier (n = 2) and one each of Broholmer, Chinese Crested Dog, Dachshund, Havanese Dog, Italian Greyhound, Labrador, mixed breed, Parson Jack Russell, Shetland Sheepdog, Slovensky Cuvac, and Staffordshire Terrier. Indications for CT examination were suspected neoplastic disease (n = 7), suspected portosystemic shunt (n = 2), suspected but excluded perineal hernia (n = 1), back pain (n = 1), immune‐mediated disease (n = 1), and gastrointestinal disease (n = 1).

In the retrospective study, CT protocols varied based on clinical indication among routinely used protocols in VTHUH. The slice and interval thicknesses were 0.625 mm, except in one dog where 1.25 mm was used in the scan. Tube potential was 100–140 kV depending on the dog's size. The collimation pitch was 0.516 or 0.984:1, speed 20.62 or 39.37 mm/rotation, and rotation time 0.4–0.8 s. The tube current varied between 80 and 602 mA depending on the dog's size and the noise index. The noise index varied between 10 and 20. The technical parameters of the CT scans of each dog are presented in Supplementary Table 1. Because of the characteristics of the retrospective study, anesthesia was not standardized. Contrast medium (Omnipaque^®^ 300 mg/ml, Oslo, Norway, GE Healthcare) was administered intravenously into the vena cephalica at a dosage of 2 ml/kg and flow rate of approximately 2 ml/s using a power injector (Medrad^®^ Stellant CT Injection System, Leverkusen, Germany, Bayer AG). The time delay between administration and image acquisition varied (portal or interstitial phase) depending on the indication for performing CT.

In the prospective study, 10 male intact cadavers were collected. The average ± SD (range) age and body weight were 9.2 ± 2.8 (2.2–13.2) years and 18.7 ± 14.3 (1.5–37.6) kg, respectively. The dogs presented nine different breeds: mixed breed (n = 2) and one each of Border Terrier, Chihuahua, Dalmatian, German Shepherd, Pomeranian, Russian Toy, Smooth Fox Terrier, and White Shepherd. The reasons for euthanasia were one each of gallbladder disease, hemorrhagic gastroenteritis, periodontal disease, shoulder arthrosis, and unspecified (n = 6). Most dogs were scanned the day of euthanasia (n = 4) or the day after euthanasia (n = 3) and the rest (n = 3) at 4, 6, or 8 days after euthanasia.

### 3.2 Volume

In the retrospective study, the average ± SD (range) CT volume of all measurements of the 13 prostate glands was 34.6 ± 30.6 (1.6–111.9) cm^3^. The average volumes of the 13 prostate glands measured by observers #1 and #2 were 34.7 ± 30.6 (1.6–110.8) cm^3^ and 34.5 ± 30.5 (1.6–111.9) cm^3^, respectively. The average volumes in the noncontrast and contrast groups, measured by both observers, were 34.5 ± 30.4 (1.6–110.8) cm^3^ and 34.7 ± 30.7 (1.6–111.9) cm^3^, respectively.

In the prospective study, the average ± SD (range) CT volume of prostate glands was 22.0 ± 17.8 (1.4–52.7) cm^3^ when all measurements of the ten prostates were included. The average CT volume measurements of ten cadavers from images acquired by five different CT protocols, protocols #1 to #5, were 21.6 ± 17.7 (1.4–52.0) cm^3^, 21.8 ± 17.8 (1.5–52.3) cm^3^, 21.8 ± 17.7 (1.5–52.4) cm^3^, 21.7 ± 17.7 (1.5–52.2) cm^3^, and 23.5 ± 17.8 (1.6–52.7) cm^3^, respectively. The average CT volume measurements of eight cadavers from images using different slice thicknesses, from 0.625 mm to 2.5 mm, were 24.4 ± 18.6 (1.5–52.2) cm^3^, 24.3 ± 18.5 (1.4–51.2) cm^3^, and 24.3 ± 18.5 (1.2–51.3) cm^3^. The average ± SD (range) volume of ten cadavers measured by the water displacement method was 21.7 ± 17.3 (1.5–50.0) cm^3^.

### 3.3 Intraobserver evaluation

According to the one‐way ANOVA model, repeatability was 99.91–99.98% of perfect agreement between repeated volume measurements of the 13 prostates in all four groups within observers and within noncontrast and contrast groups. Moreover, intraobserver ICC values showed almost perfect agreement between repeats in all groups (Table 2). Almost perfect agreement remained after combining the groups, first both observers together and then the noncontrast and contrast groups together (Table 3).

**TABLE 2:** Intraobserver agreement using intraclass correlation coefficients (ICCs) within noncontrast and contrast groups of image series

Observer #	Number of dogs	Contrast	ICC^a^	95% CI
1	13	noncontrast	0.999	0.998–1.000
1	13	contrast	0.999	0.998–1.000
2	13	noncontrast	1.000	0.999–1.000
2	13	contrast	1.000	0.999–1.000

Abbreviation: CI, confidence interval

^a^ Intraclass correlation coefficients using an absolute agreement definition.

**TABLE 3:** Agreement within noncontrast and contrast groups when measurements of both observers and finally all measurements were combined

Group	Number of dogs	ICC^a^	95% CI
noncontrast	26	1.000	0.999–1.000
contrast	26	0.999	0.999–1.000
all measurements	52	1.000	0.999–1.000

Abbreviations: ICC, intraclass correlation coefficients; CI, confidence interval.

^a^ Intraclass correlation coefficients using an absolute agreement definition

### 3.4 Interobserver evaluation

According to the one‐way ANOVA model, the repeatability was 99.9% and 100.0% of perfect agreement between the average values of each observer's measurements in both the noncontrast and contrast groups, respectively. Interobserver ICC values showed almost perfect agreement in the noncontrast and contrast groups and in the combined data (Table 4).

**TABLE 4:** Interobserver agreement using intraclass correlation coefficients (ICCs), first within noncontrast and contrast groups and finally for full data

Group	Number of dogs	ICC^a^	95% CI
noncontrast	13	1.000	0.999–1.000
contrast	13	1.000	1.000–1.000
all measurements	26	1.000	0.999–1.000

Abbreviation: CI, confidence interval

^a^ Intraclass correlation coefficients using an absolute agreement definition

### 3.5 Evaluation between different CT protocols and slice thicknesses

**FIGURE 3:**
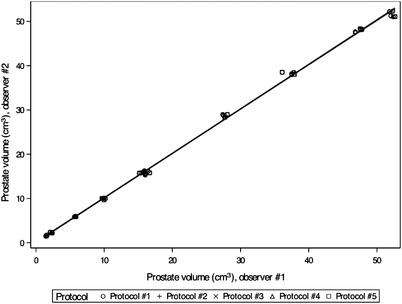
Scatter plot of linear regression analysis to demonstrate the agreement of the two observers. Measurements performed from all image series acquired using five different CT protocols were included. The regression line has a slope of 1.004 (0.993 to 1.014) and an intercept of 0.115 (‐0.192 to 0.421). On the x‐axis, prostate volumes (cm^3^) measured by observer #1 and on the y‐axis, prostate volumes (cm^3^) measured by observer #2 are shown

**FIGURE 4:**
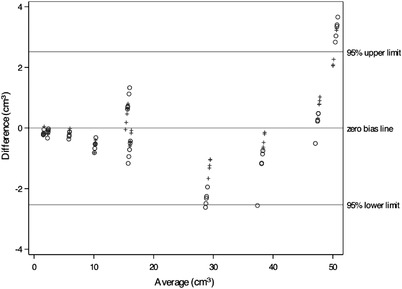
Bland–Altman diagram to demonstrate the difference between CT volume and water displacement method volume against the average of these two measurements. Markers o and + are used for the measurement of observers #1 and #2, respectively, and each of five CT protocols are represented with identical markers. A solid horizontal line with a *y*‐axis intercept of 0 represents perfect agreement, and the average bias is located at the same level. The 95% LoAs are also displayed as solid horizontal lines. The y‐axis shows the difference between the two paired measurements, and the *x*‐axis represents the average of these measurements

According to a linear mixed effects model, no significant differences emerged between volume measurements from images acquired by five different CT protocols. In Figure 3, where all CT protocols were included, a simple linear regression analysis showed almost perfect agreement between the two observers. When these volumes were compared pairwise against the water displacement method using a Bland–Altman plot, there was no consistent bias of one method versus the other. Over 90% of the measurements were situated within the 95% LoA, with upper and lower limits of ± 2.5 cm^3^ (Figure 4). Furthermore, according to a linear mixed effects model, no significant differences were shown in CT volumes between different slice thicknesses.

**FIGURE 5:**
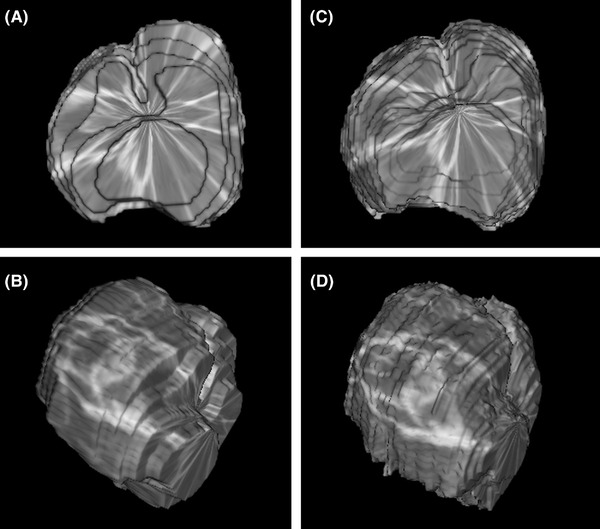
3D ROIs from computed tomography images of a cadaver prostate demonstrating measured volumes. ROIs were drawn on the image series reconstructed with a soft tissue algorithm. On the left (A and B), 2.5 mm thick slices and on the right (C and D), 0.625 mm thick slices were used. On the top (A and C) transverse view, dorsal recumbency, caudal aspect. On the bottom (B and D), oblique view from the same 3D ROI as on the top. ROI; region of interest, defined with a freehand image tracing software tool

Figure 5 presents 3D renditions of one prostate to demonstrate the variation of shape when using different slice thicknesses, 0.625 mm and 2.5 mm. This visually detected variation did not cause a significant difference in measured volume.

## 4 DISCUSSION

In our study, intra‐ and interobserver analysis and evaluation between different CT protocols and slice thicknesses supported our hypothesis, which assumed that measuring prostate CT volume using the OsiriX^®^ software tool is reliable, repeatable, and not influenced by observer, CT protocol, or contrast enhancement. Additionally, when the prostate CT volume was compared with the reference volume acquired by the water displacement method, agreement without consistent bias between the methods was shown.

Intraobserver variation in prostate CT volume measurement has not previously been evaluated in either veterinary or human studies. However, intraobserver variability in orbital CT volume has been assessed in human studies. In one study, the authors found that the average difference between repeated measurements was smaller than 5%.^23^ In another study, highly reproducible results were reported when the same images of the orbit were re‐evaluated by the same observer two weeks after the initial assessment.^19^ In our study, intraobserver variability in CT volume measurements was minimal. Based on our results, one measurement is sufficient for evaluating prostate CT volume.

Additionally, interobserver variability was minimal. Earlier studies of interobserver variability in CT volume measurements have mostly been in human medicine using several different software.^16^, ^17^, ^18^ When two observers independently measured the volumes of eight abdominal organs on the same patient's CT scan, average differences were less than 5% for all organs, except adrenal glands. Researchers suggested that small measurement errors in volumes were amplified for smaller organs (e.g., right adrenal 6% and left adrenal 21%).^16^ Interobserver variability was also evaluated by assessing human liver CT volumes.^17^, ^18^ In one study, two trained medical students and a specialized liver radiologist measured the liver volumes of 25 patients using two different software programs.^17^ In another study, two newly trained and two experienced observers measured the liver CT volumes of 30 patients with two software programs.^18^ In these studies, interobserver variability was small within and between software, and the level of experience did not significantly affect the results.^17^, ^18^ Similarly, a study assessing interobserver variability of orbital volumes measured by two surgeons demonstrated a high level of accuracy.^19^ These findings are consistent with our results of minimal interobserver variability. In addition, a clinician familiarized with the procedure and local anatomy is capable of taking accurate measurements, which are comparable to measurements made by an experienced veterinary radiologist.

Based on our review of the literature, no previously published studies were found comparing volumes measured on images acquired using different CT protocols or on both noncontrast and contrast images of the same scan. Typically, the measurements have been done on contrast image series.^12^, ^16^, ^17^, ^18^, ^21^ We found that volume measurements of the prostate do not differ between images acquired using different protocols or between noncontrast and contrast images. Based on these results, noncontrast images or images acquired using different protocols can be used and are comparable to each other for clinical or research purposes.

In veterinary medicine, the effect of slice thickness has not been previously reported based on our review of the literature. We found that a slice thickness varying between 0.625 and 2.5 mm did not significantly affect CT volume measurements, which is consistent with previous literature in human medicine.^20^, ^21^ Studies assessing the effect of slice thickness using human prostate or liver volumes report that accuracy decreases when slice thicknesses of over 5 mm were used. In addition, smaller objects were significantly more affected by slice thickness than larger ones.^20^, ^21^ Volume measurement using small slice thickness is quite time‐consuming and would therefore not be practical in routine clinical use. In our study, we did not routinely record the required time for CT measurements. However, a medium‐sized prostate with a volume of 50 cm^3^ and extending to an area of 70 slices took approximately 15 minutes to measure when 0.625 mm slices were used. When the slice thickness increased to 2.5 mm, the time needed was less than 4 minutes. We noted that slice thickness could be increased to at least 2.5 mm without sacrificing accuracy. Considering this finding and the previous literature, with larger objects, it might be time efficient to increase slice thickness somewhat, accepting the risk of mildly decreased accuracy. Furthermore, with OsiriX^®,^ it is possible to outline only part of the slices manually, afterwards outlining the rest of the slices by using the tool ‘generate missing ROIs’; this is a useful and time‐saving method.^17^ Another time‐saving method to calculate the volume of the prostate is to use the ellipsoid formula and linear measurements of the prostate, including length, width, and height. However, in one study, it was found that the ellipsoid formula method underestimates the prostate CT volume between 22.4–31.1% when compared with volume measured using a similar technique to OsiriX^®^.^28^

In the prospective part, there was no consistent bias of the CT volume versus water displacement method, and over 90% of measurements were situated within the 95% LoA (Figure 4). However, some outliers were detected. The outliers were probably due to the accuracy of the graduated cylinder. When large prostates were measured, a graduated cylinder with a grading division of 5 cm^3^ had to be used. We assume that this led to lower measurement accuracy in the water displacement method and caused larger differences when compared with CT volumes. Only a few previous studies have compared CT volumes with actual reference volumes. ^12^, ^14^, ^15^ Our results are consistent with these earlier studies. Instead of actual volume, several volumetric studies have used different reference methods, such as other diagnostic imaging modalities or resection weights. In these studies evaluating the human liver and prostate, overestimation of CT volumes was reported.^17^, ^24^, ^25^, ^26^ However, the cause of the overestimation remained unclear, and it was not necessarily due to the CT volume measurement itself. Suspected causes for overestimation were changes in blood volume after resection,^17^, ^25^ differing resection lines between CT and surgery, variable specific gravity when using resection weights,^24^, ^25^ and different window levels used when comparing measurements between two software programs.^17^ When comparing CT volumes of the canine prostate to actual volumes, overestimation has not been found,^12^ which is consistent with our results. Changes in fluid content are possible during both resection and sinking of the preparation in the water during measurement with the water displacement method. This was not noticed in our study. The effect of varying the window level causing potential overestimation of the measured volume was excluded in our study by standardizing the window level.

Difficulties in defining prostate boundaries and contouring the prostate on CT images have been reported in human medicine. Examples of these are the tendency to include portions of neurovascular bundles, poor definition of the interface between the posterior prostate edge and the anterior rectal wall, and difficulties in distinguishing the lower limit of the prostate apical region because of its close proximity to the pelvic floor muscles and the poor contrast between these two soft tissues.^27^ In our material, we did not have similar difficulties. However, we excluded dogs with known prostatic disease, which was presumably why we did not have severely altered prostates in our sample. The ability to contour prostate boundaries might be different with enlarged and irregularly shaped prostates that protrude close to adjacent soft tissues. The small and regularly shaped prostates in our study were surrounded by fat tissue, which gave good contrast to the prostate tissue and helped to define the prostate boundary even without contrast medium.

Algorithms of the volume measurement tools of different software are not generally open to users, making direct comparisons difficult. In our study, no concerns were brought forth that our findings could not also be generalized to other software, which provide a similar technique for volume measurement. The advantages of OsiriX^®^ include that it is not bounded to the workstation of the CT scanner, and it is easily available for users. A potential obstacle for users who want to use OsiriX^®^ is that it is only compatible with the Mac^®^ operating system.

This study has several limitations. First, because we excluded dogs with known prostatic disease, the results cannot be generalized to severely abnormal and enlarged prostates. Second, the number of dogs and cadavers was small. Third, the graduated cylinder with a grading division of 1–5 cm^3^ enables a maximum measurement accuracy of one decimal, while in CT measurements, four decimals were calculated. Thus, the water displacement method provides only a coarse reference to which the CT volume can be compared. Fourth, comparisons between different protocols, slice thicknesses, and CT volume and water displacement methods were performed only with cadavers. Finally, all protocol parameters and conditions could not be standardized in the retrospective study, e.g., the acquisition time of contrast images varied.

In conclusion, measuring the prostate volume on CT images is a repeatable and reliable method. Volume measurements have sufficient accuracy for clinical and research purposes regardless of which CT protocol is used in the acquisition of images. Additionally, measuring the volume from noncontrast images is as repeatable and reliable as measuring the volume from contrast images. The reliability of the measurements is not dependent on the availability of a specialist; a clinician familiar with the technique is also capable of measuring the prostate. Further studies are needed to evaluate the reliability of volume measurements of severely abnormal or enlarged prostates and to determine reference values for prostate size on CT.

## LIST OF AUTHOR CONTRIBUTIONS

### Category 1

(a) Conception and Design: Salonen, Åhlberg, Laitinen‐Vapaavuori, Mölsä
(b) Acquisition of Data: Salonen, Åhlberg
(c) Analysis and Interpretation of Data: Salonen, Åhlberg

### Category 2

(a) Drafting Article: Salonen, Åhlberg, Laitinen‐Vapaavuori, Mölsä
(b) Revising Article for Intellectual Content: Laitinen‐Vapaavuori, Mölsä

### Category 3

(a) Final Approval of the Completed Article: Salonen, Åhlberg, Laitinen‐Vapaavuori, Mölsä

### Category 4

(a) Agreement to be accountable for all aspects of the work ensuring that questions related to the accuracy or integrity of any part of the work are appropriately investigated and resolved: Salonen, Åhlberg, Laitinen‐Vapaavuori, Mölsä

## CONFLICT OF INTEREST

The authors have declared no conflict of interest.

## PREVIOUS PRESENTATION OR PUBLICATION DISCLOSURE

There is no previous presentation or publication.

## REPORTING GUIDELINE DISCLOSURE

The GRRAS checklist for reporting of studies of reliability and agreement was used for this manuscript.

## Supporting information

Supporting Information
