# Supplementary material for: CT measurement of prostate volume using OsiriX® viewer is reliable, repeatable, and not dependent on observer, CT protocol, or contrast enhancement in dogs
Source: Vet Radiol Ultrasound. 2022 Jul 5;63(6):729–38. doi: 10.1111/vru.13125 (PMC9795897; doi:10.1111/vru.13125)
Supplement: Supplementary file 1 — Supporting Information [file VRU-63-729-s001.docx]

**Supplementary Table 1****.** Technical parameters used in the CT scan of each dog, including the caudal abdomen, in the retrospective study.

| Dog # | Slice thickness (mm) | Voltage (kV) | Pitch | Speed (mm/rot) | Rotation time (s) | Current (mAs) | Contrast phase |
| --- | --- | --- | --- | --- | --- | --- | --- |
| 1 | 1.25 | 140 | 0.984:1 | 39.37 | 0.5 | 325–602 | interstitial |
| 2 | 0.625 | 120 | 0.516:1 | 20.62 | 0.8 | 80–120 | interstitial |
| 3 | 0.625 | 120 | 0.984:1 | 39.37 | 0.5 | 100 | portal |
| 4 | 0.625 | 120 | 0.984:1 | 39.37 | 0.5 | 100 | portal |
| 5 | 0.625 | 120 | 0.984:1 | 39.37 | 0.5 | 133–386 | portal |
| 6 | 0.625 | 120 | 0.984:1 | 39.37 | 0.4 | 248–399 | interstitial |
| 7 | 0.625 | 120 | 0.984:1 | 39.37 | 0.5 | 302–475 | interstitial |
| 8 | 0.625 | 120 | 0.516:1 | 20.62 | 0.6 | 87–110 | interstitial |
| 9 | 0.625 | 100 | 0.516:1 | 20.62 | 0.8 | 98–104 | interstitial |
| 10 | 0.625 | 120 | 0.984:1 | 39.37 | 0.5 | 211–273 | portal |
| 11 | 0.625 | 100 | 0.516:1 | 20.62 | 0.8 | 101–102 | interstitial |
| 12 | 0.625 | 120 | 0.984:1 | 20.62 | 0.8 | 248–311 | portal |
| 13 | 0.625 | 120 | 0.984:1 | 39.37 | 0.8 | 80 | interstitial |
